# Editorial: Problematic Gaming, Personality, and Psychiatric Disorders

**DOI:** 10.3389/fpsyt.2019.01004

**Published:** 2020-01-17

**Authors:** Vladan Starcevic, Yasser Khazaal

**Affiliations:** ^1^Faculty of Medicine and Health, Sydney Medical School, Nepean Clinical School, Discipline of Psychiatry, University of Sydney, Sydney, NSW, Australia; ^2^Department of Psychiatry, Nepean Hospital, Penrith, NSW, Australia; ^3^Addiction Medicine, Department of Psychiatry, Lausanne University Hospitals, Lausanne, Switzerland; ^4^Faculty of Biology and Medicine, Lausanne University, Lausanne, Switzerland; ^5^Research Center, Montreal University Institute of Mental Health, Montreal, QC, Canada

**Keywords:** gaming, internet gaming disorder, gaming disorder, personality, psychiatric disorder

Problematic gaming has been receiving increasing attention in recent years from both clinicians and researchers. This largely explains the introduction of two novel diagnostic categories: Internet gaming disorder (IGD) in The Diagnostic and Statistical Manual of Mental Disorders, 5th Edition (DSM-5) and gaming disorder (GD) in The International Classification of Diseases, 11th Revision (ICD-11). Both diagnoses have elicited controversies that boil down to two fundamental questions. First, should some patterns of problematic gaming be conceived of as a mental disorder? Second, what are the relationships between problematic gaming and psychopathology? The Research Topic “Problematic Gaming, Personality, and Psychiatric Disorders” was proposed to address the second issue. Most of the eight articles from this Research Topic that were published in *Frontiers in Psychiatry* have approached this issue from different perspectives, and they are briefly reviewed in this Editorial.

Any research in this area crucially depends on the quality of measurement instruments. An article by Laconi and colleagues focused on the assessment of a measure of problematic Internet use—a broad construct of particular relevance for problematic gaming. The authors reported good psychometric properties of the nine-item Problematic Internet Use Questionnaire (PIUQ-9), used for screening of problematic Internet use. The study was conducted in a large number of participants in nine European countries, thus supporting the use of the PIUQ-9 across various European languages and cultures.

In their article “Internet Gaming Disorder in Adolescents with Psychiatric Disorder: Two Case Reports Using a Developmental Framework,” Benarous and colleagues showed the value of the developmental approach to problematic gaming. They identified early attachment problems as playing a pivotal role in the two contrasting pathways to problematic patterns of gaming. One pathway was labelled “internalized” and encompassed social anxiety and avoidant behaviors; the other pathway was “externalized,” as it was characterized by impulsivity and poor emotion regulation. The take-home message is that problematic gaming is not a homogeneous entity and that its treatment entails an effort to understand the specific antecedent developmental factors and target the corresponding maladaptive personality characteristics and psychopathology.

A variety of personality traits has been linked with problematic gaming, with many of these relationships apparently being nonspecific. Laier et al. investigated the relationships between IGD symptoms, maladaptive personality traits, and gaming-related cognitions, i.e., expectancies. Not surprisingly, the authors reported that various personality traits, as well as gaming-related positive and gaming-related avoidance expectancies, were related to the symptoms of IGD. However, the relationship between IGD symptoms and specific personality traits, i.e., negative affectivity, detachment, and psychoticism, was mediated by avoidance expectancies, which pertain to the notion that gaming helps to escape from reality and avoid or reduce negative feelings. These findings shed more light on the nature of these relationships, clarifying the role of certain personality traits, as well as the different roles played by different gaming-related expectancies.

While the relationship between attention deficit/hyperactivity disorder (ADHD) and problematic gaming is well established (1), its interpretation and understanding have been elusive, largely because of the paucity of longitudinal data. Marmet et al. studied a very large sample of young Swiss men and examined longitudinal associations over a 5-year span. They reported that ADHD increased the risk of GD and that GD increased the risk of ADHD. Importantly, these associations pertained more to the inattention component of ADHD than to the hyperactivity component of ADHD. These findings strongly suggest a need for screening and early diagnosis and treatment of ADHD and GD in individuals who present primarily with GD and ADHD, respectively.

A study from South Korea by Seong et al. was one of the few conducted in individuals seeking hospital treatment for their gaming. In this group of presumably more severely ill gamers, depressed mood and symptoms of ADHD, especially inattention, were found to lead to treatment-seeking in the hospital. This underscores the clinical significance of ADHD and depressive symptoms in problematic gaming.

The relationship between problematic gaming and depression was the subject of an article by Liu and colleagues. These authors investigated neural mechanisms underpinning the relationship between IGD and depression in college students and reported that after psychotherapy intervention, the amygdala-frontoparietal connectivity did not contribute to a decrease in depressive symptoms co-occurring with IGD. This led to a suggestion that aberrant resting-state functional connectivity between emotion and executive control networks might underlie depression in IGD and that this should be specifically targeted in the course of treatment.

Emotion dysregulation has been frequently postulated as an underlying and/or transdiagnostic factor in a variety of psychopathological manifestations (2). In an important, large study of emotion regulation strategies in problematic adolescent gamers, Kökönyei and colleagues reported that all maladaptive emotion regulation strategies were associated with problematic gaming. This finding is not surprising, but it suggests that such emotion regulation strategies could potentially be targeted in the treatment of problematic gaming.

Gambling disorder is the only other behavioral addiction officially recognized as a mental disorder and included in both DSM-5 and ICD-11. The links between IGD/GD and gambling disorder have been important for conceptualizing problematic gaming as a mental disorder. More recently, these links have been reflected in a trend to combine online gaming and gambling, for example, *via* loot boxes (3). Therefore, the Research Topic also included an article by Jiménez-Murcia and colleagues that examined various phenotypes of gambling disorder using a clinical clustering analysis. The authors reported three mutually exclusive groups of patients with gambling disorder, characterized by high, moderate, and mild emotional distress, respectively. These levels of distress were associated with different severity of co-occurring psychopathology, possibly reflecting different subtypes of gambling disorder. It would be interesting to apply the same methodology to IGD/GD in an effort to ascertain whether there are subtypes that might be responsive to different treatments.

The articles included in the Research Topic “Problematic Gaming, Personality, and Psychiatric Disorders” highlight the importance of screening and assessing co-occurring personality traits and psychopathology among individuals with problematic gaming. While some of these relationships are nonspecific and reflect transdiagnostic phenomena such as negative affectivity, emotion dysregulation, avoidant behaviors, and impulsivity, others are relatively specific and even more relevant for treatment. Any effort to help and treat individuals with problematic gaming should be adapted to take into account findings of these articles.

## Author Contributions

Both authors participated in the preparation of this article and approved its final version.

## Conflict of Interest

The authors declare that the research was conducted in the absence of any commercial or financial relationships that could be construed as a potential conflict of interest.
